# Nutritional Stress and Prey Signals Trigger a Metabolic Shift in *Arthrobotrys* spp.: *Aomae1* Expression Suggests a Role in the Switch Between Saprophytic and Parasitic Lifestyles

**DOI:** 10.3390/pathogens15050519

**Published:** 2026-05-12

**Authors:** María José Hernández-Vega, Pedro Mendoza-de Gives, David Emanuel Reyes-Guerrero, Gustavo Pérez-Anzúrez, Génesis Andrea Bautista-García, Edgar Jesús Delgado-Núñez, Agustín Olmedo-Juárez, Ana Yuridia Ocampo-Gutiérrez, María Eugenia López-Arellano, Elke von Son-de Fernex

**Affiliations:** 1 Laboratory of Helminthology, National Centre for Disciplinary Research in Animal Health and Safety (CENID-SAI), National Research Institute for Forestry, Agriculture and Livestock (INIFAP), Jiutepec 62574, Morelos, Mexico; majovega306@gmail.com (M.J.H.-V.); pedromdgives@yahoo.com (P.M.-d.G.); reyes.david@inifap.gob.mx (D.E.R.-G.); tavopzaz@gmail.com (G.P.-A.); bagg150583@gmail.com (G.A.B.-G.); olmedo.agustin@inifap.gob.mx (A.O.-J.); yumiga2002@yahoo.com.mx (A.Y.O.-G.); 2 Department of Research, Polytechnic University of Morelos State (UPEMOR), Boulevard Paseo Cuauhnahuac No. 566, Colonia Lomas del Texcal, Jiutepec 62550, Morelos, Mexico; 3 Faculty of Agricultural, Livestock and Environmental Sciences, Autonomous University of the State of Guerrero, Iguala de la Independencia 40040, Guerrero, Mexico; edgarjezus@gmail.com; 4 Teaching, Research and Extension in Tropical Livestock Center, Faculty of Veterinary Medicine and Zootechnics, National Autonomous University of Mexico (CEIEGT-FMVZ-UNAM), Martínez de la Torre 93600, Veracruz, Mexico

**Keywords:** nematode-trapping fungi, *Aomae1* gene, biological control, liquid culture filtrates, nutritional stress, gastrointestinal nematodes

## Abstract

Nematode-trapping fungi are saprophytic organisms that can transform their mycelium into a parasitic lifestyle, forming traps to capture and feed on nematodes. Although this transition is triggered by environmental conditions, the genetic regulation of this metabolic shift remains unclear. This study assessed the effects of nutritional stress on mycochemical synthesis, trap morphogenesis, and *Aomae1* gene expression in *Arthrobotrys oligospora* and *Arthrobotrys musiformis*. Fungal biomass was subjected to the following three-stage successive culture model: (i) nutrient-rich (Czapek–Dox broth), (ii) nutritional stress (water), and (iii) media enriched with live prey (*Haemonchus contortus* Hc-L_3_). Samples were taken for molecular analysis, and liquid culture filtrates (LCFs) were recovered for chromatographic identification of mycochemical groups. To assess trap formation (traps/cm^2^), mycelia from each culture model was transferred to water agar plates and defied with Hc-L_3_. Results showed a significant bioenergetic trade-off. Both starvation and larval presence induced a downregulation of mycochemical synthesis, which resulted in the total loss of nematocidal activity in LCfs, while triggering a morphogenetic response. *Arthrobotrys musiformis* showed the most aggressive phenotype with 3.8-fold increase in trap formation and a massive 429.05-fold overexpression of *Aomae1* under predatory challenge. While *A. oligospora* showed a similar but less pronounced trend (2.4-fold increase in trap formation and 44.48-fold *Aomae1* overexpression), our findings suggest that *Aomae1* expression plays a critical role in the metabolic switch that regulates and redirects energy resources, prioritizing mechanical trapping mechanisms over secondary metabolism during nutrient scarcity. These findings highlight *Aomae1* as a possible key activator for virulence, which offers strategic targets for the optimization of biocontrol agents against gastrointestinal nematodes in livestock.

## 1. Introduction

*Haemonchus contortus* (*Nematoda: Trichostrongylidae*) is a blood-sucking parasitic nematode living in the stomach of small ruminants and causes severe damage to the health and productive potential of the flocks all over the world [1]. Conventional treatment to control haemonchosis is based on the frequent administration of chemically synthesized anthelmintic drugs. However, its use in the livestock industry raises concerns associated not only to the presence of anthelmintic resistance [2] but also to the global concern regarding the presence of chemical residues in the products and sub-products destined for human consumption [3] and the ecotoxicity associated with drug elimination into the environment through feces and urines [4]. Therefore, to counteract the negative impact of chemical use, other methods such as biological control have been explored as an alternative. Among the most assessed biological control agents against nematodes, we can find bacteria [5], acari [6], predatory nematodes [7], and nematophagous fungi [8]. Nematode-trapping fungi (NTF) are microorganisms that can switch between saprophytic and parasitic lifestyles, feeding on both dead organic matter and living nematodes [9]. *Arthrobotrys* spp. is one of the most studied genera of NTF. It was first described by Zopf in 1888 and has been widely studied as a potential biological control agent [10] against nematodes of importance in both agriculture [11,12] and livestock [13]. *Arthobotrys oligospora* and *A. musiformis* are two of the main species of NF that have been assessed for their predatory activity and their ability to produce bioactive compounds and enzymes with nematocidal activity [14,15,16,17,18]. The transition of NF from its saprophytic to predatory stage has been directly associated with interactions with nematode species, inducing morphogenesis and the expression of virulence genes in these fungi [19]. In recent years, advances in proteomics have identified genes associated with the NF infection process, including host recognition, adhesion, penetration, and digestion. In this regard, the Peroxin *Pex14/17* gene has been directly associated with key biological activities of *A*. *oligospora,* including trap formation, pleiotropic roles, mycelial development, stress response, and secondary metabolism [20]. It has also been reported that the disruption of *277* and *279* genes in *A. oligospora* is associated with the production of polyketide–terpenoid hybrid metabolites and an increase in fungal nematocidal ability [21]. Furthermore, the *Aomae1* gene has been associated with lipid droplet accumulation, conidiation, trap formation, and the production of secondary metabolites like arthrobotrisins [22]. It has been reported that environmental conditions, such as osmotic and oxidative stress, as well as changes in luminosity and nutritional factors, among others, affect the growth, development, and pathogenicity of NTF [23]. Thus, the objectives of this research were to assess the effect of nutritional stress on the relative expression of the *Aomae1* gene in *A*. *oligospora* and *A*. *musiformis*, and to examine its association with the production of secondary metabolites with anthelmintic-like and predatory activity.

## 2. Materials and Methods

### 2.1. Location

This study was carried out at the National Center of Disciplinary Research in Animal Health and Innocuity (CENID-SAI) belonging to the National Institute of Research in Forestry, Agriculture and Livestock (INIFAP), (Agricultura) from the Mexican Government at Jiutepec City, Morelos, Mexico.

### 2.2. Biological Material and Fungal Liquid Cultures (FLCs)

#### 2.2.1. Nematode-Trapping Fungi (NTF) Isolates

The NTF used were *Arthrobotrys oligospora* (GenBank: PP567275) and *A. musiformis* (GenBank: PP333206), both belonging to the fungal collection of the Laboratory of Helminthology of CENID-SAI-INIFAP. The isolates *A. oligospora* and *A. musiformis* were originally recovered in prior studies [24,25] from soil samples from a poultry farm (Cuernavaca City, Morelos, Mexico) and from a private orchard (Cuautla City, Morelos, Mexico), respectively. Both fungi were produced in water agar plates and maintained at 23.6 ± 2.7 °C for 15 days. Both isolates were taxonomically identified following the morphometric descriptions published by Oorschot De Hoog (1985) [26].

#### 2.2.2. Haemonchus Contortus Infective Larvae

*Haemonchus contortus* (*Nematoda: Trichostrongylidae*) infective larvae (Hc-L_3_) were obtained from a donor lamb with a mono-specific infection (isolate Hc-Hueytamalco, CENID-SAI-INIFAP). Biological material recovery and lamb housing were performed as described by Colinas-Picazo [24]. The lamb management complied with the Norma Official Mexicana (Official Mexican Standard) with the official rule number NOM-052-ZOO-1995 (http://www.senasica.gob.mx, accessed on 8 August 2023), as well as with the Ley Federal de Sanidad Animal (Federal Law for Animal Health) DOF DOF 07-06-2012, following the ethical standards outlined by INIFAP.

#### 2.2.3. Fungal Nutritional Stress Assay

Both fungal isolates (*A*. *oligospora* and *A*. *musiformis*) were subjected to the following three-stage successive culture model: (i) nutrient-rich (Czapek–Dox broth), (ii) nutritional stress (starvation in water), and (iii) media enriched with live prey (water with 10^4^ *Haemonchus contortus* (*Nematoda: Trichostrongylidae*) infective larvae (Hc-L_3_)). Initially, each isolate was individually cultured in 250 mL flasks containing 50 mL of Czapek–Dox broth (CzDxB) (Sigma-Aldrich, Saint Louis, MO, USA) for 21 days under static conditions at room temperature (23.6 ± 2.7 °C). The control group (CzDxB) was maintained under identical conditions throughout all successive incubation stages. Three replicates were run for each isolate, treatment, and control. Following the initial stage incubation, the fungal biomass was recovered, weighed, and 200 mg of mycelial biomass was collected into 2 mL tubes to perform RNA extraction. To induce nutritional stress (second stage), 2 g fresh weight of the remaining mycelial biomass was transferred to a flask containing 50 mL of sterile distilled water and incubated for 7 days. At the end of this starvation period, another biomass sample (200 mg) was collected for molecular analysis. Finally, for the third stage, 1 g fresh weight from the remaining biomass was transferred into a flask containing 50 mL of sterile distilled water, nutritionally enriched with 10^4^ live *H. contortus* infective larvae (Hc-L_3_*),* and incubated for another 7-day period. Afterward, the last sample of mycelial biomass (200 mg) was collected for molecular analysis. Furthermore, the liquid culture filtrates (50 mL) obtained after each incubation period were recovered and stored separately for further chromatographic identification of the mycochemical groups.

#### 2.2.4. Fungal Liquid Culture Filtrates

The fungal liquid cultures (LCFs) for each treatment were individually filtered to obtain sterile LCFs containing the secondary metabolites. To separate the mycelial biomass from the liquid medium, a series of filtrations was performed using different materials as follows: a coffee filter paper, a Whatman paper # 4 (25 μm pore diameter) (Merck^®^, KGaA, Darmstadt, Germany), a 2 μm glass fiber filter (Millipore^®^, KGaA, Darmstadt, Germany), and finally, the liquid material was passed through two consecutive filtrations using a 0.45 μm and 0.22 μm nitrocellulose filters, respectively (Millipore^®^, KGaA, Darmstadt, Germany). Fungal liquid cultures were then recovered and concentrated using a rotatory evaporator (Büchi R-300, Flawil, Switzerland) for subsequent freeze-drying (Labconco^®^, Kansas, MO, USA). Once lyophilized, the FLCs were stored at 4 °C until use for chromatographic identification of mycochemical groups and to assess nematocidal activity.

### 2.3. Nematocidal Activity of LCFs and Fungal Isolates

#### 2.3.1. Nematocidal Activity of Liquid Culture Filtrates (LCFs)

The freeze-dried fungal liquid cultures (15 mg) obtained from each isolate (*A. oligospora* and *A. musiformis*) and each treatment were individually reconstituted with 1.5 mL of either water or phosphate-buffered saline solution (PBS) to obtain a stock solution of 10 mg/mL. Final concentrations obtained through serial dilutions were: 5 mg/mL, 2.5 mg/mL, 1.25 mg/mL and 0.625 mg/mL. The LCFs treatments assessed were: (1) liquid culture of the isolate grown in CzDxB, (2) CzDxB without fungi as a control, (3) liquid culture of the isolate under stress conditions (water), (4) liquid culture after nutrient enrichment (Hc-L_3_), and (5) water (negative control). The interaction larvae/liquid culture filtrates were performed in 96-well microtiter plates. Fifty microliters of an aqueous suspension containing approximately 100 Hc-L_3_ (50 μL) were placed on wells of the microtiter plate (*n* = 3), and 50 μL of the corresponding LCFs treatment was added and incubated at room temperature (24.7 ± 0.6 °C) for 72 h. After the incubation period, 50% of the total volume from each well was analyzed to estimate the number of live and dead larvae. This was done by counting the larvae in ten 5 μL aliquots on a slide. To distinguish between live and dead larvae, we applied a physical stimulus by gently touching their cuticle with a needle. Larvae that remained motionless in response to this stimulus were classified as dead. Live and dead larvae were then counted, and the percentage of larval mortality per treatment was calculated [24].

Larvae found dead after being exposed to the fungal liquid culture filtrates were transferred onto a slide for observation under a Leica Zeiss DM6B microscope (Leica Zeiss^®^, Wetzlar, Germany) using the objectives 10×, 20×, and 40×. We monitored any possible larval morphological changes resulting from the effects of the liquid culture filtrates. Additionally, a set of microphotographs were taken using a microscope camera and the LAS Program (v4.9).

#### 2.3.2. Assessment of the Trap Formation of Isolates Pre- and Post-Nutritional Stress

Following each step of the three-stage successive culture model (see Section 2.2.3), a 200 mg (fresh weight) sample of mycelium was transferred from each flask to water agar plates with a 6 cm diameter (*n* = 12; treatments and control) and incubated for 7 days at room temperature (23.6 ± 2.7 °C). Each plate was then inoculated with 100 µL of an aqueous suspension containing 200 ensheathed live *H. contortus* L_3_. Plates were kept at room temperature (24.14 ± 0.9 °C) for additional 7 days. Following this period, three random 1 cm^2^ areas on the agar surface were observed under a microscope at 4 and 10× magnifications to count the number of traps. For this purpose, the plate bases were previously gridded and numbered, and three squares (1 cm^2^) were randomly selected for counting using a computer true random number generator (random.org). The mean number of traps produced per treatment was then estimated.

### 2.4. Mycochemical Screening of LFCs Using Thin Layer Chromatography (TLC)

The screening of mycochemical groups present in the different LCFs was performed through thin layer chromatography (TLC) using the following reagents and methods: (i) the Dragendorff Mayer and Wagner’s reagents were used to identify the presence of alkaloids; (ii) the Bornträger test was used to identify the presence of coumarins; (iii) Mg^2+^ (magnesium ion) and HCl (hydrochlorhydric acid) tests were used to determine the presence of flavonoids; (iv) the presence of tannins was evidenced through the ferric chloride 10% (FeCl_3_) gelatine and saline solution tests; and (v) triterpenes and sterols were identified following the Lieberman–Burchard and Salkowski reaction [27]. The qualitative assessment of these mycochemical groups was performed through direct observation, evaluating both the intensity and the rate of color development, and were categorized as follows: (i) (−) negative: no color change, (ii) (+) slightly positive: a slight, slow color change and (iii) (++) positive: a distinct, moderately fast color change, and (iv) (+++) strong positive: intense, fast color change [28,29].

### 2.5. Molecular Techniques

#### 2.5.1. Fungal RNA Extraction

The RNA purification was performed using TRIzol^®^, a commercial reagent (Thermo Fisher Scientific, Waltham, MA, USA), following the manufacturer’s instructions. Firstly, 0.5 g of a fungal mycelia sample was disrupted four times into a microtube (D1030, Merk, Rahway, NJ, USA, 2 mL) with 500 µL of Trizol and 1.0 mm Zirconium beads (Merck Z7663780) for homogenization at 4000 rpm at 40 s in a homogenizer (Benchmark Scientific, South Plainfield, NJ, USA). In each interval, tubes were placed into an ice plastic box for 45 s to avoid an excess of heat in the samples. Afterwards, the homogenized liquid was transferred into a 2 mL tube and centrifuged at 4 °C for 5 min at 12,000× *g* to collect the supernatant in a 1.5 mL with 100 μL of chloroform, and it was softly hand-mixed and centrifuged for 15 min at 4 °C and at 12,000× *g*. In a new 2 mL tube with 50 µL of isopropanol and the supernatant containing the aqueous phase, it was incubated at room temperature (23.6 ± 2.7 °C) for 10 min, after which it was centrifuged for 10 min at 4 °C at 12,000× *g*. The supernatant was discarded by decantation and the pellet conserved to add 500 μL of ethanol (at 75%) in Eppendorf tube to homogenize and separate the pellet. Then, the sample was centrifuged at 7500× *g* at 4 °C for 5 min, the supernatant was discarded by decantation, and total RNA in the pellet was recovered. The tube containing the pellet was placed unevenly on desiccant paper for 15 min to allow drying of residual ethanol droplets present. The pellet was re-hydrated with 50 μL of free-nuclease water and incubated at 57 °C for 10 min, the RNA concentration and purity were determined using a nanophotometer (IMPLEN^®^, Munich, Germany), and the total relationship between RNA purity and concentration was recorded at an absorbance ratio of 260/280 nm.

#### 2.5.2. Fungal cDNA Synthesis and RT-qPCR Assays

The cDNA synthesis was performed following the RT PROMEGA Improm-II Reverse Transcription System (Madison, WI, USA), following the manufacturer’s instructions and the protocol cited by [30]. After cDNA synthesis, the purity and concentration were recorded using a nanophotometer previously (relationship A260/280). The cDNA synthesis used was at 300 ng using Oligo dT primers, following the manufacturer’s instructions and the protocol described by [31]. For the qPCR assays, we used *Aomae1* gene of interest and *β-tubulin* as the housekeeping gene. The qPCR assays were performed in quadruplicate for each gene in this study. Primers were previously designed using the sequences XP_011119752 for *Aomae1* and XM_011123192 for *β-tubulin*, BLASTN, Primer-BLAST, and OligoAnalyzer software (NCBI BLAST+ v2.16.0, 2024). The qPCR reaction for each gene was performed at a final volume of 20 μL in 0.2 mL microtubes. The RT-qPCR reaction mixture contained GoTaq RT-qPCR Master Mix (PROMEGA, Madison, WI, USA), 20 μM (1 μL) of each primer, and 500 ng of cDNA. The primers used for *Aomae1* amplification were 5′F: TGGCCGCTACATTCGGTATC and 3′R: TGGTTGGTAGGTAACGCCAC. The primers for *β-tubulin* amplification were 5′F: ACGGCTCCGGTGTTTACAAT and 3′R: GACCCTTCGCCCAGTTGTTA. The RT-qPCR cycling conditions were as follows: initial denaturation at 95 °C for 5 min, followed by 40 cycles of denaturation at 95 °C for 10 s, annealing at 62 °C for 20 s, and extension at 72 °C for 20 s. The qPCR assays were performed on a Rotor-Gene 6000 thermocycler (Corbett Research^®^, Hilden, Germany).

### 2.6. Statistical Analysis

The comparative Ct method was used to perform (in triplicate) the relative expression analysis of *Aomae1* gene in both *Arthrobotrys* spp. isolates, based on the number of cycles at which the amplification plot crossed the threshold. Raw Ct values were recorded from each assay under the three nutritional conditions. The Ct values of the *Aomae1* target gene and the housekeeping gene were subtracted to normalize the mRNA levels, providing a relative expression value using the 2^ΔΔCt^ method. Each technical replicate of each gene from both control and experimental samples was analyzed through the GeneGlobe platform (https://geneglobe.qiagen.com/us/analyze, accessed on 10 May 2024) (Qiagen^®^, Venlo, The Netherlands), which automatically applies a statistical correction for multiple comparisons [30]. The Student’s *t*-test (*p* = 0.05) was used to evaluate significant differences using the 2^ΔΔCt^ values.

Data obtained from the nematocidal activity bioassay of LCFs were analyzed using Kruskal–Wallis test for independent samples (α = 0.05), with each condition evaluated separately based on fungus, concentration and culture condition factors. This analysis compared larval mortalities across each condition, using the same concentration and the same fungal culture. Fungi were compared using the Mann–Whitney U test for each individual culture condition and concentration (*p* < 0.05). For total trap formation (traps/cm^2^), data from both fungi across the three different culture conditions were analyzed using the ANOVA followed by the Tukey test for pairwise comparisons of conditions within each fungus (α = 0.05).

## 3. Results

### 3.1. Taxonomic Identification of Both Fungal Isolates

The results of the taxonomic identification, based on morphology and morphometry of both fungal isolates, are presented in Table 1.

**Figure 1:**
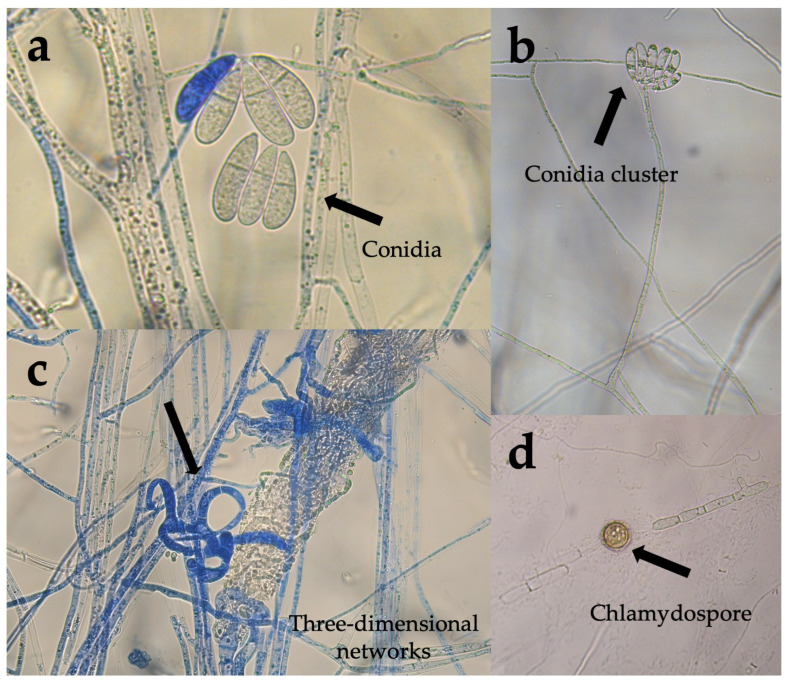
Microphotographs taken with a Leica DM6 upright optical microscope showing some structures of taxonomic importance typical of *Arthrobotrys musiformis*. (**a**) Elongated conidia clusters; (**b**) conidiophore crowned by a conidia cluster; (**c**) three-dimensional adhesive nets; (**d**) chlamydospore.

**Figure 2:**
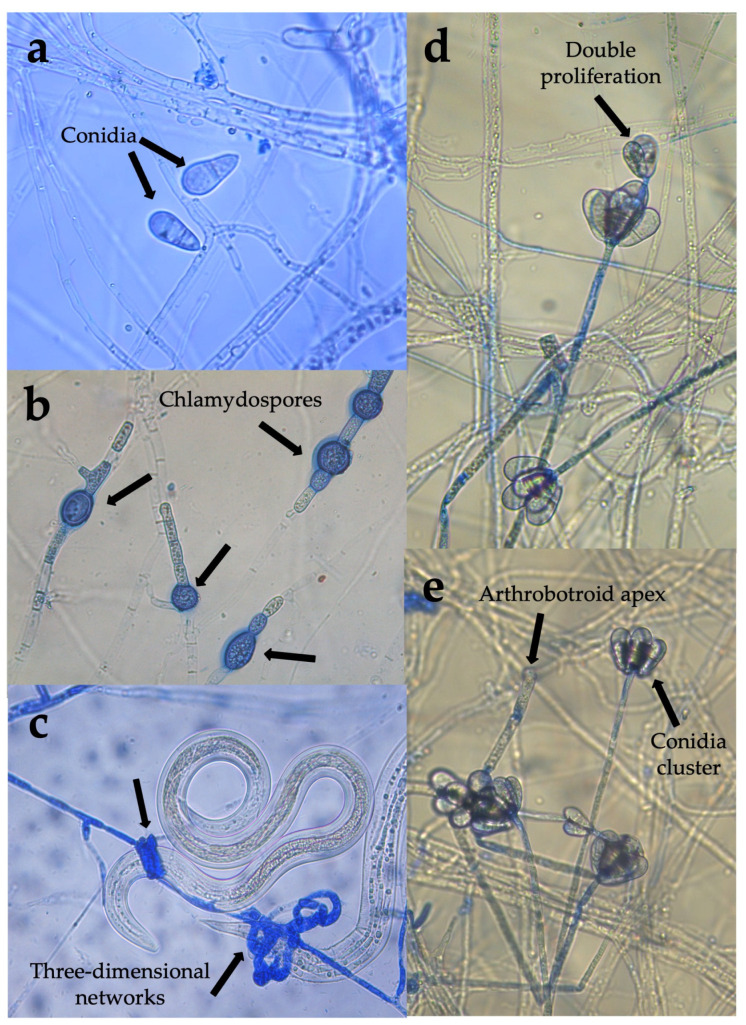
Microphotographs taken with a Leica DM6 upright optical microscope showing some structures of taxonomical importance typical of *Arthrobotrys oligospora*. (**a**) Obovoidal septate conidia; (**b**) chlamydospores; (**c**) three-dimensional adhesive nets and (**d**) double proliferation of the conidiophore and (**e**) conidia clusters and an arthrobotryoid apex conidiophore.

Some of the main morphological features corresponding to aerial structures of both isolates (*Arthrobotrys musiformis* and *A. oligospora*) are shown in Figure 1 and Figure 2, respectively. The first isolate analyzed showed the presence of hyaline hyphae, bicellular conidia and simple, erect and non-branched conidiophores. Conidia showed repeated proliferations and an obovoidal to piriform aspect, with a light constriction at septum slightly below the middle. Conidiophores were crowned with up to 10 conidia clusters. These features matched with those recorded for *Arthrobotrys oligospora* (Figure 1). Likewise, the other isolate showed conidia longer than *A. oligospora*. Conidiophores showed a candelabrum-like branching system (candelabrelloid apex), where conidia are produced. Conidia were elongated-obovoidal to ellipsoidal straight or slightly curved. Conidiophores were erect and long and single. Chlamydospores were present. This fungus traps nematodes using three-dimensional adhesive nets. These features corresponded to the species *A. musiformis* (Figure 2).

### 3.2. Nematocidal Activity of Fungal Liquid Culture Filtrates

At the highest concentration tested (5 mg/mL) in CzDxB, the LCFs of both nematophagous fungi exhibited strong nematocidal activity. *Arthrobotrys oligospora* reached a larval mortality of 94.83% (IQR: 86.40–97.08%; U = 269, *p* = 0.026), significantly higher than that of *Arthrobotrys musiformis* 56.41% (IQR: 22.93–93.55%). However, nematocidal activity of LCFs derived from the nutritional stress (starvation) and media enriched with live prey (10,000 HcL_3_) was completely lost in both isolates when compared to CzDxB (H(df = 2) = 3.041, *p* > 0.05). Mortality observed in the water control groups was minimal (2.65 ± 0.69 and 3.05 ± 0.74 for *A. musiformis* and *A. oligospora*, respectively).

### 3.3. Trap Formation

**Figure 3:**
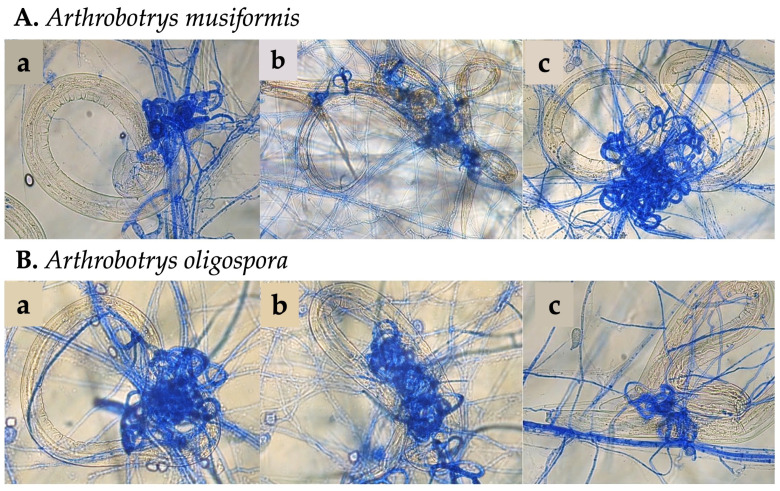
Three-dimensional adhesive traps. (**A**) *Arthrobotrys musiformis* and (**B**) *A. oligospora*; after being subjected to a three-stage successive culture model: (**a**) nutrient-rich (CzDxB), (**b**) nutritional stress (starvation in water), and (**c**) media enriched with live prey (water with 10^4^ Hc-L_3_).

Both *Arthrobotrys* spp. isolates showed a strong induction of trap formation (trap/cm^2^) in response to the nutrient availability (Figure 3). Nutritional stress (starvation in water) did not induce a significant increase in trap formation for either isolate (*p* > 0.05). However, the medium was enriched with live prey (10,000 HcL_3_) and the predatory activity showed a 2.4- and 3.8-fold increase for *A. oligospora* and *A. musiformis*, respectively. The averages of counted traps per cm^2^ are shown in Table 2.

### 3.4. Analysis of Mycochemical Groups

For both isolates, the LCFs with higher concentration and diversity of mycochemical groups were the ones obtained from the nutrient-rich (CzDxB) culture media. The mycochemical groups observed on each LCFs obtained from the three-stage successive culture model are presented in Table 3.

### 3.5. Relative Expression Analysis

The relative expression of the *Aomae1* gene was significantly influenced by nutrient availability in both isolates. In the nutritional stress (starvation), *A. oligospora* showed a slight 1.82-fold induction in *Aomae1* expression, while *A. musiformis* isolate showed a significant basal induction of 26.32-fold (*p* < 0.05), suggesting that in this strain, *Aomae1* is more susceptible to nutrient availability. However, in the presence of the prey (water +Hc-L_3_), gene expression was drastically induced in both fungal isolates up to 44.48-fold (*p* < 0.01) and 429.05-fold increase (*p* < 0.001) for *A. oligospora* and *A. musiformis*, respectively. The reference gene, β-tubulin, remained stable in all treatments. The results of the relative expression analysis of *Aomae1* are shown in Table 4.

## 4. Discussion

Sustainable control strategies such as the use of nematode-trapping fungi (NTF) and fungal liquid culture filtrates (LCFs) have been explored as an alternative for parasite control management due to the increasing emergence of anthelmintic resistance [32]. Maximizing both predatory and nematocidal activity of fungi has been a focal point of novel research to increase their effectiveness under field conditions [25,33]. Nematode-trapping fungi are facultative saprobic organisms and their transition to a predator stage is often triggered by nutritional stress [23]. Understanding the impact of nutrient availability on both secondary metabolite production and virulence of nematophagous fungi (NF), and the role of gene expression like *Aomae1*, could be critical for optimizing biological control efficacy against gastrointestinal nematodes (GINs) in grazing cattle.

The most outstanding observation in this project was the metabolic plasticity of *Arthrobotrys* spp. exposed to successive nutritional shifts. We were able to capture the exact transition between the saprotrophic phase (characterized by mycochemical synthesis) and the predatory phase (defined by increased trap formation and downregulation of the secondary metabolism). Our data identify *Aomae1* gene to have a critical regulatory role for the bioenergetic trade-off involved in this biological process; further studies of functional validation are suggested to confirm its role in the switch between saprophytic and parasitic lifestyles.

In alignment with prior reports [24,25,34], LCFs from *A. oligospora* and *A. musiformis* in nutrient-enriched media (Czapek–Dox) achieved larval mortality rates of 90% and 55%, respectively. This nematocidal activity was associated with the presence of mycochemicals like alkaloids, coumarins, saponins and/or tannins/isocumarins. Even though the full biological activity of NTF secondary metabolites remains debated, prior reports have suggested that mycochemicals are secreted by NTF either as: (i) chemical defense (to inhibit or kill other microorganisms, against mycophagous organisms, or even to monopolize nutrient resources), (ii) as specialized molecules favoring fungi fitness before it transitions to a predacious lifestyle, or (iii) as protection against environmental conditions [14,35,36,37,38].

The nutritional stress (transferred to water) triggered the metabolic shift in both *Arthrobotrys* spp., stopping the energy-expensive production of mycochemicals to prioritize trap formation for the mechanical attack of nematodes [39,40]. Interestingly, upon exposure to live *H. contortus* infective larvae (Hc-L_3_), a positive reappearance of coumarins in *A. oligospora* LCF and slightly positive reappearance of coumarins, tannins and alkaloids in *A*. *musiformis* LCF suggests that while nutrient deprivation suppresses mycochemical synthesis, the larval chemical signal is powerful enough to reactivate specific biosynthetic pathways, even in a nutrient-limited media, emphasizing the targeted nature of the fungal defense mechanism.

The link between *Aomae1* gene and the secondary metabolism showed a negative correlation in our isolates, with a significant reduction in mycochemical synthesis, while gene overexpression achieved 44.48-fold and 429.05-fold for *A*. *oligospora* and *A*. *musiformis*, respectively. The decreased production of mycochemicals is consistent with Liu et al. (2023) [22] who reported that *Aomae1* gene is responsible for both up- and down-regulation of over 474 mycocompounds. Furthermore, our data partially differ from the statement that *Aomae1* depletion in mutant strains results in a reduction in mycochemical production [20], as our results show a significant overexpression (26.32-fold) when secondary metabolites were at the lowest, and massive overexpression (429.05-fold in *A*. *musiformis*) when mycochemical levels were low. This suggests that *Aomae1* gene is critical for predatory mechanisms and represents an important synchronizing genetic component to downregulate the isolates’ secondary metabolism, which has a highly energetic cost, and to redirect that energy towards trap formation, a metabolic strategy that ensures a biologically successful transition into a predatory stage when nutrients are scarce and prey is present.

The differences observed between isolates LCFs AH-like activity, mycochemical synthesis, virulence, and *Aomae1* expression, even when cultured under the same nutritional stress/challenge conditions, aligns with an inter-species variability regarding predatory activity and secondary metabolism, previously reported for *Arthrobotrys* spp. [41]. Furthermore, it shows that *Arthrobotrys musiformis* has a much more aggressive genetic and morphogenetic response than *A*. *oligospora*, suggesting a more specialized predatory nature and highlighting isolate-specific metabolic strategies. This implies that the *A*. *musiformis* strain is metabolically primed for secondary metabolite synthesis, a crucial factor when selecting strains for industrial biocontrol application [24].

## 5. Conclusions

Our findings demonstrate that mycochemical (secondary metabolites) and physical (traps) components of predation in *A*. *oligospora* and *A*. *musiformis* are synchronized through a trade-off mechanism. *Aomae1* overexpression showed a strong involvement in the genetic switch controlling the transition from saprobic to predatory stage, being highly sensitive to both nutritional deprivation and prey signaling. While mycochemical synthesis is energy-dependent, and isolates cultured under nutrient-rich conditions favor nematocidal activity of LCFs, this study provides molecular foundation for a strategic selection of NTF strains and the optimization of culture media to enhance biological control strategies against gastrointestinal nematodes in livestock.

## Figures and Tables

**Table 1:** Morphometric characteristics of *Arthrobotrys musiformis* and *Arthrobotrys oligospora*.

Morphometric Characteristics	*Arthrobotrys musiformis*	*Arthrobotrys oligospora*
Conidial shape	Ovoid to ellipsoid, smooth-walled and transparent, i.e., hyaline. Slightly curved, with the distal end wider and rounded.	Obovoid to pyriform and slightly constricted at the septum. Its conidia are less elongated than those of *A. musiformis*.
Conidium length (μm)	29.05 (25.78–39.15)	22.45 (18.95–23.56)
Conidium width (μm)	11.95 (11.05–15.65)	10.22 (9.82–12.40)
Septa	1	1
Conidiophore	Conidiophores erect, simple and sometimes branched. They end in clusters of conidia that appear more elongated or linear.	Long and erect conidiophores with the gradual and progressive formation of denticles along its structure, from which clusters of conidia are generated, formed by two cells separated by a septum.
Conidiophore length (μm)	257.07 (135.48–405.02)	378.50 (193.42–453.30)
Chlamydospores	Present	Present
Type of trap	Three-dimensional adhesive nets	Three-dimensional adhesive nets

**Table 2:** Trap formation obtained from both isolates (*Arthrobotrys musiformis* and *A. oligospora*) per cm^2^ on the agar surface, after subjected to the following three-stage successive culture model: (i) nutrient-rich (CzDxB), (ii) nutritional stress (starvation in water), and (iii) media enriched with live prey (water with 10^4^ Hc-L_3_).

Culture Condition	Traps/cm^2^ (Mean + SD)	CI (95%)	Ratio
*Arthrobotrys musiformis*			
Nutrient-rich (CzDxB)	2.17 ± 0.4 ^a^	1.59–2.74	1
Nutritional stress (starvation)	3.57 ± 0.8 ^a^	2.96–4.17	1.6
Live prey (water + Hc-L_3_)	8.33 ± 2.6 ^b^	6.45–10.21	3.8
*Arthrobotrys oligospora*			
Nutrient-rich (CzDxB)	5.56 ± 1.2 ^a^	4.1–7.02	1
Nutritional stress (starvation)	6.10 ± 2 ^a^	4.65–7.55	1.1
Live prey (water + Hc-L_3_)	13.43 ± 5.3 ^b^	9.64–17.23	2.4

Different letters among rows within each fungus species represent statistically significant differences (*p* < 0.05). Estimated as the relation between traps counted in water or water + HcL_3_ and CzDxB. (*n* = 3).

**Table 3:** Mycochemical profile of filtrates obtained from *Arthrobotrys musiformis* and *A. oligospora* strains under three-stage successive culture model.

Mycochemicals	*Arthrobotrys musiformis*			*Arthrobotrys oligospora*		
	CzDxB	Starvation	Prey HcL_3_	CzDxB	Starvation	Prey HcL_3_
Alkaloids	++	++	+	+	−	−
Coumarins	++	−	+	++	−	++
Flavonoids	−	−	−	−	−	−
Tannins	++	−	+	−	−	−
Triterpenes + sterols	−	−	−	−	−	−
Saponins	−	−	−	+	−	−

(−) negative: no color change; (+) slightly positive: a slight, slow color change; (++) positive: a distinct, moderately fast color change.

**Table 4:** Relative expression analysis of the *Aomae1* gene from two nematophagous fungi, *Arthrobotrys musiformis* and *A. oligospora*, under the three-stage successive culture model.

	*A. musiformis*		*A. oligospora*	
	*Aomae1*	*β*-Tubulin	*Aomae1*	*β*-Tubulin
	2^−ΔCT^-Fold Change			
CzDxB	0.000179	1.00000	0.004804	1.00000
Starvation	0.004705–26.32 *	1.00–1.00 ***	0.008749–1.82	1.00–1.00 ***
(Prey Hc-L_3_)	0.07668–429.05 ***	1.00–1.00 ***	0.213652–44.48 **	1.00–1.00 ***

Control group: fungi growing in Czapek–Dox broth (CzDxB). Asterisks show statistical significance between rows and columns within a species: *** *p* < 0.001; ** *p* < 0.01; * *p* < 0.05.

## Data Availability

The original contributions presented in this study are included in the article. Further inquiries can be directed to the corresponding author.

## Abbreviations

The following abbreviations are used in this manuscript:

NTF	Nematode-trapping fungi
Hc-L_3_	*Haemonchus contortus* infective larvae
CzDxB	Czapek–Dox broth
TLC	Thin layer chromatography
RNA	Ribonucleic acid
cDNA	Complementary deoxyribonucleic acid
RT-qPCR	Reverse transcription quantitative polymerase chain reaction
GINs	Gastrointestinal nematodes
AH-like	Anthelmintic-like

## References

[1] Strydom T., Lavan R., Torres S., Heaney K. (2023). The economic impact of parasit-ism from nematodes, trematodes and ticks on beef cattle production. Animals.

[2] Borges F.D.A., Amarante A.F.T.D., Lopes W.D.Z., Canton C., Alvarez L., Lifschitz A. (2024). Anthelmintic resistance of gastrointestinal nematodes in cattle in Brazil and Argentina-current status and global perspectives. Rev. Bras. Parasitol. Vet..

[3] Montalvo R.R., Alcívar E.Z. (2024). Impacto de residuos de ivermectina en los alimentos de origen animal: Revisión. Rev. Cient. Arb. Multidisc. PENTACIENC.

[4] Verdú J.R., Cortez V., Rosa-García R., Ortiz A.J., García-Prieto U., Lumaret J.P., Sánchez-Piñero F. (2023). Nontoxic effects of thymol, carvacrol, cinnamaldehyde, and garlic oil on dung beetles: A potential alternative to ecotoxic anthelmintics. PLoS ONE.

[5] Imani Baran A. (2023). A mini-review of Bacillus thuringiensis application to control important economic and zoonotic parasites. J. Zoonot. Dis..

[6] Dos Anjos K.A., Duarte F.C., Katiki L.M., Giglioti R., Santos B.G., Mendes M.C. (2024). In vitro evaluation of the potential of mites of the family *Macrochelidae* (*Acari: Mesostigmata*) as macrobiological agents against the nematode *Haemonchus contortus* (*Strongylida: Trichostrongylidae*). Vet. Parasitol..

[7] da Silva M.E., Mercado M.A., Millán-Orozco J., Mendoza de Gives P., Liébano Hernández E., Ribeiro Braga F., de Araújo J.V. (2017). Predatory activity of Butlerius nematodes and nematophagous fungi against *Haemonchus contortus* infective larvae. Rev. Bras. Parasitol. Vet..

[8] Fernández S., Zegbi S., Sagües F., Iglesias L., Guerrero I., Saumell C. (2023). Trapping behavior of *Duddingtonia flagrans* against gastrointestinal nematodes of cattle under year-round grazing conditions. Pathogens.

[9] Berhanu M., Gebeyaw D.T., Kefale D., Kang Y. (2024). Overview of nematophagous fungi, isolation techniques, and their role in biological control of helminthic parasites: A literature review. Acta. Entomol. Zool..

[10] Niu X.-M., Zhang K.-Q. (2011). *Arthrobotrys oligospora*: A model organism for understanding the interaction between fungi and nematodes. Mycology.

[11] Rodríguez-Esquivel D.L., Ocampo-Gutiérrez A.Y., Olmedo-Juárez A., López-Arellano M.E., Hernández-Romano J., Aguilar-Marcelino L., Mendoza-de Gives P. (2024). Using *Arthrobotrys oligospora* (Orbiliales) spores mixed with sterile sheep faeces for disinfesting soil micro-plots infested with *Nacobbus aberrans* (Nematoda: Pratylenchidae). Biocont. Sci. Technol..

[12] Wang D., Ma N., Rao W., Zhang Y. (2023). Recent advances in life history transition with nematode-trapping fungus *Arthrobotrys oligospora* and its application in sustainable agriculture. Pathogens.

[13] Szewc M., De Waal T., Zintl A. (2021). Biological methods for the control of gastrointestinalnematodes. Vet. J..

[14] Degenkolb T., Vilcinskas A. (2016). Metabolites from nematophagous fungi and nematicidal natural products from fungi as an alternative for biological control. Part I: Metabolites from nematophagous ascomycetes. Appl. Microbiol. Biotechnol..

[15] Júnior A.D., Ferreira V.M., de Carvalho L.M., Álvares F.B.V., Vilela V.L.R., Ferraz C.M., Veloso F.B.R., Lima T.F., Braga F.R., de Araújo J.V. (2021). Association of the nematophagous fungi *Arthrobotrys musiformis* and *Monacrosporium sinense* in vitro and in vivo for biological control of equine cyathostomins. Braz. J. Vet. Med..

[16] Purba R.T.T., Fauzi F., Sari R.W., Naibaho D.C., Putri Q.A., Maulana A., Punnapayak H. (2022). *Arthrobotrys thaumasia* and *Arthrobotrys musiformis* as biocontrol agents against *Meloidogyne hapla* on tomato plant. Biodiversitas.

[17] Li S., Wang D., Gong J., Zhang Y. (2022). Individual and combined application of nematophagous fungi as biological control agents against gastrointestinal nematodes in domestic animals. Pathogens.

[18] Jaramillo-Tlalapango J., Mendoza-de Gives P., Higuera-Piedrahita R.I., Ocampo-Gutiérrez A.Y., López-Arellano M.E., Pérez-Anzúrez G., Olmedo-Juárez A., Hernández-Romano J., Maza-López J., Delgado-Núñez E.J. (2023). Study of a Mexican isolate of *Arthrobotrys musiformis* (*Orbiliales*): Predatory behavior and nematocidal activity of liquid culture filtrates against *Haemonchus contortus* (*Trichostrongylidae*), protein profile and myco-constituent groups. Fungal Biol..

[19] Kuo C.Y., Tay R.J., Lin H.C. (2024). The nematode-trapping fungus *Arthrobotrys oligospora* detects prey pheromones via G protein-coupled receptors. Nat. Microbiol..

[20] Liu Q., Li D., Bai N., Zhu Y., Yang J. (2023). Peroxin Pex14/17 is required for trap formation, and plays pleiotropic roles in mycelial development, stress response, and secondary metabolism in *Arthrobotrys oligospora*. Msphere.

[21] Chen Y.H., Liu X., Dai R., Ou X., Xu Z.F., Zhang K.Q., Niu X.M. (2020). Novel polyketide-terpenoid hybrid metabolites and increased fungal nematocidal ability by disruption of genes 277 and 279 in nematode-trapping fungus *Arthrobotrys oligospora*. J. Agri. Food. Chem..

[22] Liu Y., Zhu M., Wang W., Li X., Bai N., Xie M., Yang J. (2023). *AoMae1* Regulates Hyphal Fusion, Lipid Droplet Accumulation, Conidiation, and Trap Formation in *Arthrobotrys oligospora*. J. Fungi.

[23] Bohórquez S.M.A., Rico R.O.G. (2019). Efecto de diferentes condiciones de estrés sobre el crecimiento vegetativo del hongo filamentoso *Acremonium chrysogenum*. BISTUA Rev. Fac. Cienc. Bás..

[24] Colinas-Picazo A., Mendoza-de Gives P., Pérez-Anzúrez G., Gutiérrez-Medina E., Bautista-García G.A., Delgado-Núñez E.J., Olmedo-Juárez A. (2024). Assessing the In Vitro Individual and Combined Effect of *Arthrobotrys oligospora* and *A. musiformis* (*Orbiliales*) Liquid Culture Filtrates against Infective Larvae of the Sheep Blood-Feeding Nematode *Haemonchus contortus* (*Trichostrongylidae*). Pathogens.

[25] Pérez-Anzúrez G., Olmedo-Juárez A., von-Son de Fernex E., Alonso-Díaz M.Á., Delgado-Núñez E.J., López-Arellano M.E., Mendoza-de Gives P. (2022). *Arthrobotrys musiformis* (*Orbiliales*) kills *Haemonchus contortus* infective larvae (*Trichostronylidae*) through its predatory activity and its fungal culture filtrates. Pathogens.

[26] Oorschot C.V. (1985). Taxonomy of the Dactylaria complex, V. A review of *Arthrobotrys* and allied genera. Stud. Mycol..

[27] Pérez-Anzúrez G., Mendoza-de Gives P., Alonso-Díaz M.Á., von Son-de Fernex E., Paz-Silva A., López-Arellano M.E., Olmedo-Juárez A. (2024). *Lecanicillium psalliotae* (Hypocreales: *Cordycipitaceae*) exerts ovicidal and larvicidal effects against the sheep Blood-Feeding nematode *Haemonchus contortus* through its liquid culture filtrates. Pathogens.

[28] Rivas-Morales C., Oranday-Cárdenas M.A., Verde-Star M.J. (2016). Investigación en Plantas de Importancia Médica.

[29] Harborne J.B. (1998). Phytochemical Methods: A Guide to Modern Techniques of Plant Analysis.

[30] Cedillo-Borda M., López-Arellano M.E., Reyes-Guerrero D.E. (2020). In vitro assessment of ivermectin resistance and gene expression profiles of P-glycoprotein genes from *Haemonchus contortus* (L3). Bio-Protocol.

[31] Reyes-Guerrero D.E., Higuera-Piedrahita R.I., Maza-Lopez J., Mendoza-de-Gives P., Camas-Pereyra R., López-Arellano M.E. (2024). Analysis of P-gp genes relative expression associated to ivermectin resistance in *Haemonchus contortus* larval stages from in vitro cultures (L3 and xL3) and from gerbils (*Meriones unguiculatus*) (L4) as models of study. J. Helmint..

[32] Faria L.E.M., dos Santos Fonseca J., de Araújo J.V., de Carvalho L.M., Albuquerque G.R., de Souza Perinotto W.M. (2025). Nematophagous fungi to controlling gastrointestinal nematodes in small ruminants: A systematic review. Vet. Parasitol..

[33] de la Crúz-Crúz H.A., Higuera-Piedrahita R.I., Zamilpa A., Alcalá-Canto Y., Ocampo-Gutiérrez A.Y., Arango-de la Pava L.D., Mendoza-de Gives P. (2025). Using an Aqueous Suspension of *Duddingtonia flagrans* Chlamydospores and a Hexane Extract of *Artemisia cina* as Sustainable Methods to Reduce the Fecal Egg Count and Larvae of *Haemonchus contortus* in the Feces of Periparturient Ewes. Pathogens.

[34] Bahena-Nuñez D.S., Ocampo-Gutiérrez A.Y., Mendoza-de Gives P., González-Cortázar M., Zamilpa A., Higuera-Piedrahita R.I., Hernández-Romano J. (2024). *Arthrobotrys oligospora* (Fungi: *Orbiliales*) and its liquid culture filtrate myco-constituents kill *Haemonchus contortus* infective larvae (Nematoda: *Trichostrongylidae*). Biocont. Sci. Technol..

[35] Rahman M.U., Zhong X., Uzair M. (2024). Application of fungi as biological control strategies for nematode management in horticultural crops. Phytopathol. Res..

[36] de Freitas Soares F.E., Ferreira J.M., Genier H.L.A., Al-Ani L.K.T., Aguilar-Marcelino L. (2023). Biological control 2.0: Use of nematophagous fungi enzymes for nematode control. J. Nat. Pestic. Res..

[37] Wong H.J., Mohamad-Fauzi N., Rizman-Idid M., Convey P., Aisyah Alias S. (2019). Protective mechanisms and responses of micro-fungi towards ultraviolet-induced cellular damage. Polar Sci..

[38] Künzler M. (2018). How fungi defend themselves against microbial competitors and animal predators. PLoS Pathog..

[39] Shende V.V., Bauman K.D., Moore B.S. (2024). The shikimate pathway: Gateway to metabolic diversity. Nat. Prod. Rep..

[40] Gives P.M.D., Rodriguez-Labastida M., Olmedo-Juarez A., Gamboa-Angulo M.M., Reyes-Estebanez M. (2022). A Nematode Crude Extract Acts as an Elicitor of the Nematocidal Activity of Nematophagous Fungi Liquid Culture Filtrates Against *Haemonchus contortus* (Nematoda: *Trichostrongylidae*). Acta Parasit..

[41] Wu Y., Yang Z., Jiang Z., Nizamani M.M., Zhang H., Liu M., Wei S., Wang Y., Li K. (2023). Isolation. Identification, and Evaluation of the Predatory Activity of Chinese *Arthrobotrys* Species towards Economically Important Plant-Parasitic Nematodes. J. Fungi.

